# Facilitating or hindering learning - a meta-analysis of acceleration on video learning

**DOI:** 10.3389/fpsyg.2025.1427609

**Published:** 2025-08-22

**Authors:** Guan Huang, Yixuan Du, Hairu Yang

**Affiliations:** China West Normal University, Nanchong, China

**Keywords:** speed of speech, video learning, multimedia learning, meta-analysis, playback speed

## Abstract

**Introduction:**

Time compression of instructional videos has received attention from scholars around the world. From existing empirical studies, a wide range of scholars have not yet reached a consensus on whether acceleration promotes video learning.

**Methods:**

The article adopts a meta-analytic approach to analyze 12 domestic and international experimental and quasi-experimental research papers, exploring the role of moderating variables on the effect of acceleration on learning from the dimensions of teachers’ appearances, subtitles, and subject attributes.

**Results:**

The results of the study showed that the main effect test results found that accelerated playback of instructional videos increased learners’ cognitive load (g = 0.59) and decreased maintenance test scores (g = −0.41), migration test scores (g = −0.50), and learning satisfaction (g = −0.24). Moderation analyses revealed that teacher appearances played a moderating role in accelerating the effects of cognitive load and maintenance tests, and subtitles, country, and subject attributes played a moderating role in accelerating the effects of learning satisfaction.

**Discussion:**

Therefore, instructional video designers need to proactively consider the appropriate speed when developing instructional videos, and learners need to reasonably adjust the playback speed of the videos when learning from them in order to improve the quality of video teaching and learning. For educators, when designing teaching videos, they need to fully consider the impact of publication bias on the existing research results, and avoid blindly referring to the conclusions that may overestimate the effects of acceleration.

## 1 Introduction

The rapid development of information technology has driven the iterative upgrading of the teaching paradigm. Early distance education broke the limits of time and space, allowing knowledge to be disseminated across geographical areas. Subsequently, the further development of network education, by the advantages of the Internet, achieves a more convenient interaction between teachers and students. The rise of online learning has made education accessible. Learners can independently choose the learning content and progress according to their time and needs (Liu et al., 2024). In this process, the set of sound and picture graphics in one of the educational videos plays a key role, which will be the knowledge presented vividly and intuitively, greatly enhancing the learning effect (Yang et al., 2023).

However, there is no scientific and reasonable design principle for the speed level of the current teaching video, which is made only according to the developer’s preference and lacks science, and then relies on the learner’s feelings to make up for the defect by using the multiplier playback function on their own (Feng et al., 2021). This causes learners in video learning there are some unavoidable problems, for example, because the teacher explains the speed is too slow, learners watching the video use accelerated playback or even skip some video clips (Ma et al., 2021), which leads to distracting students’ attention, learning interest is not good (De Koning et al., 2011); and the video tempo is too fast, which makes it difficult for learners to go through the video and understand it, and they need to watch the same video over and over again (Meyer et al., 2010).

The goal of an instructional designer is to maximize a learner’s comprehension and satisfaction, while minimizing the amount of time a learner will spend on a learning task (Ritzhaupt et al., 2008). Learning effect refers to the results of learning, including direct variables and indirect variables that favor learning. The direct variable is learning achievement, and in the field of learning effectiveness assessment, Mayer proposes to divide learning achievement into learning maintenance achievement and learning migration achievement, and construct a systematic measurement system. Learning maintenance focuses on learners’ ability to remember and reproduce what they have learned, and tests whether they can remember what they have learned; learning migration focuses on learners’ deeper use of knowledge, covering the construction, analysis, reasoning and practical application of the content, reflecting whether learners can flexibly use the knowledge to solve new problems, and represents a more in-depth level of learning compared with learning maintenance. Indirect variables include cognitive load and learning satisfaction (Mayer, 2006).

Integrating the multidimensional moderating variables proposed by Fiorella (2022), it was found that at the multimedia design level, accelerated playback produces a dynamic interaction with the segmentation principle and the signaling principle; at the social cues level, the degree of preservation of the teacher’s image presentation decreases as the playback speed is increased, which directly affects the social agency effect; and in the cognitive engagement dimension, the effect of generative activities shows a speed-dependent effect; For individual differences, the level of prior knowledge had a significant moderating effect. In addition, contextual factors such as content type (procedural/declarative knowledge) and auxiliary functions (subtitles, visual anchoring) together constitute a complete moderating network of video acceleration effects. Based on cognitive neuroscience research, Chinese, as an ideographic writing system, has visual symbols that directly map semantics, and this direct glyph-semantic pathway allows Chinese learners to maintain a high level of comprehension of accelerated speech in the unsubtitled condition (Zhang et al., 2022). In contrast, English relies on indirect phoneme-semantic conversion, and the speech ambiguity caused by acceleration significantly reduces comprehension accuracy (Pandey and Arif, 2022). Thus, in cross-national video learning studies, the country variable is essentially a proxy variable for differences in language perception.

Regarding the negative effects of inappropriate video playback speed on learners, many empirical studies have been devoted to exploring the question “Can acceleration promote learners’ video learning effectiveness?” Some studies have shown that accelerated playback speed has a positive impact on video learning and can improve learning efficiency and learning satisfaction (Lang et al., 2020), but some studies have come up with the exact opposite viewpoint, showing that acceleration has a significant negative impact on video learning (Pastore, 2012). In short, academics are inconclusive about the impact of accelerated playback (Pastore and Ritzhaupt, 2015). Based on this, this study adopts a meta-analytic approach to holistically evaluate the impact of the speed of instructional videos on learners’ learning outcomes, aiming to provide a scientific basis and guidance for the design, development, and application of instructional videos.

## 2 Literature review

### 2.1 Theoretical overview

Multimedia learning refers to the use of words (spoken or written) and visuals (images, animations, videos) to enhance learning (Mayer, 2014). Video learning is one of the core forms of multimedia learning and is a typical two-channel multimedia learning method. And video learning challenges learners’ cognitive systems due to its transient and linear playback characteristics. Various theories provide different explanations about the intrinsic mechanisms by which video speed affects learning outcomes.

#### 2.1.1 Cognitive Theory of Multimedia Learning

Cognitive Theory of Multimedia Learning (CTML), developed by Mayer (2006), is a foundational framework for understanding how people learn from multimedia materials, such as videos, animations, and interactive simulations. The theory is grounded in cognitive psychology and emphasizes how learners process visual and auditory information. CTML derive directly from Baddeley’s model of working memory, which Mayer inherited along with these basic assumptions, and further developed by applying them to multimedia learning situations (Baddeley, 1999).

Human working memory consists of two information processing channels, visual/auditory, which are used to process and store visual and verbal information respectively, and the storage capacity of the two channels is limited (Li et al., 2015). During video learning, learners need to process both the picture information and the speech information of the video. The information perceived by the visual and auditory senses is processed and stored separately by different regions of the brain, and the synergistic effect of multiple regions makes the memory stronger, which is conducive to the maintenance of performance. The integrated dual-channel information is more flexible and systematic, when encountering new problem situations that require knowledge migration, learners can more effectively extract and apply the knowledge to realize the migration of performance (Mayer, 2006).

#### 2.1.2 Cognitive Load Theory

Mental activity realized simultaneously with working memory is called cognitive load (Paas et al., 2004). Cognitive Load Theory (CLT) is a learning theory proposed by Australian psychologist Sweller (1988) to explain how the limited nature of human working memory affects learning efficiency. The instructional principles of the theory are based on long-term memory and working memory assumptions about human cognitive architecture. If the video speed is changed, the speed of information presentation is also changed, which will affect the cognitive load of both channels of learners (Paas et al., 2004). Acceleration makes more information enter the visual and auditory channels per unit of time, which will lead to an increase in external cognitive load and inhibit learning once the limited capacity of working memory is exceeded.

#### 2.1.3 Cognitive resource theory and perceptual load theory

Since learners need to test their learning results after video learning, the sharp decrease in learning time is likely to cause them to feel confused, anxious, and stressed, which not only reduces their learning satisfaction (Ma et al., 2021), but also negatively affects the executive control of attention, i.e., the allocation of resources (Sternberg and Mio, 2008), which is detrimental to learning. The information per unit of time increases, attention is taken up more by the learning task, the higher the perceptual load value is, the less idle attention is available, and instead, it is less likely to be disturbed (Wei and Zhou, 2005), thus promoting learning effectiveness.

**FIGURE 1:**
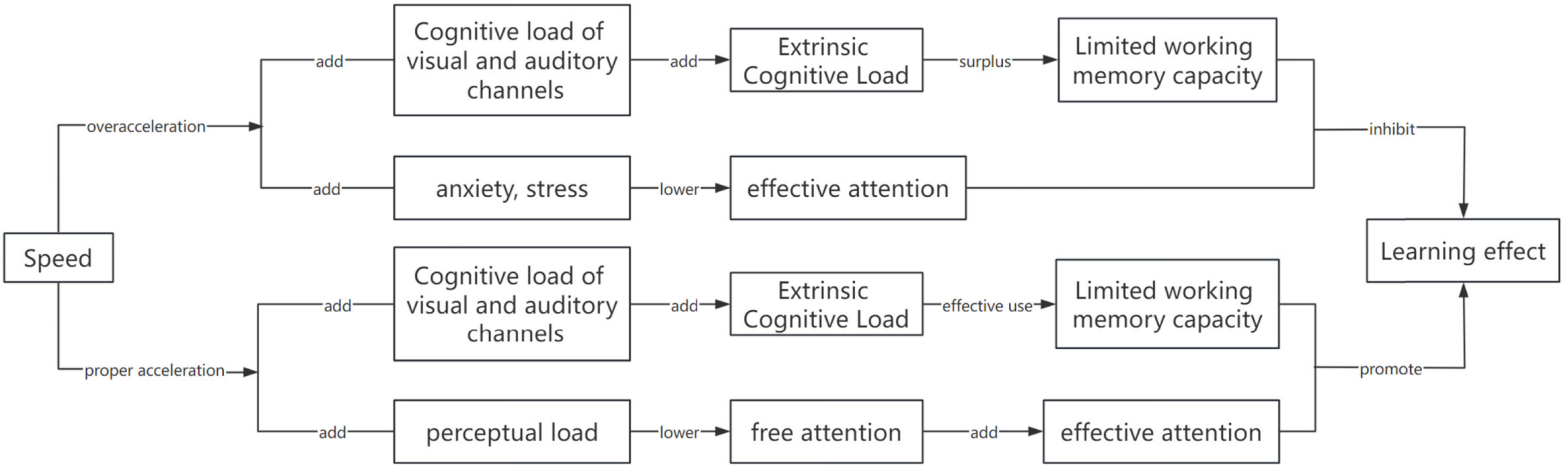
Theoretical structure of video speed affecting learning effect.

Therefore, whether acceleration can promote the learning effect of video needs to consider the impact of video on the cognitive load of learners’ visual and auditory channels, and the core issue is whether the degree of acceleration will lead to cognitive load overload and affect the emotional feelings of learners while learning. To clarify the relationship between the theories, this study attempts to construct a theoretical model of speed affecting video learning effectiveness, as shown in Figure 1.

### 2.2 Relevant studies

According to the purpose of the study, the effect of acceleration on learners’ learning effectiveness, learning satisfaction, and cognitive load were used as the outcome variables of the meta-analysis, which are summarized below around these three aspects.

Song et al. (2018) found that when learners play videos at 1.5 times the speed, it will have a certain negative impact on learning effectiveness. Feng Xiaoyan used the unsubtitled learning video “The structure of the heart and blood circulation” as the experimental material, and found that the subjects’ maintenance and migration test scores under different playback speeds were 1.25x, 1.0x, 1.5x, and that the video playback speed had a significant impact on the learning effect (Feng et al., 2020). Ma Anran utilized the hydrological cycle of the relevant MOOC as experimental materials and found that the normal group of the maintenance test was significantly higher than the 1.5x and 2x groups, and the 1.5x group was higher than the 2x group. The migration quiz normal group was higher than the 2x group. And after controlling equal video learning duration it was found that 1.5x playback does not inhibit learning compared to normal speed and 2x playback is more effective than 1.5x. In terms of learning satisfaction, learners were more satisfied with learning in normal conditions than in the 2x speed group (Ma et al., 2021). Feng Xiaoyan compared materials with different levels of difficulty and found that when the content was easier, 1.5x speed ensured learning effect; when the content was more difficult, 1.5x speed significantly reduced maintenance test scores (Feng et al., 2021). Duan Chaohui et al. used the animation of the lightning formation principle as experimental material and pointed out that in the performance of the memory test, the slow level of the migration test scores is better than the normal speed and fast (Duan et al., 2013). Ritzhaupt et al. (2015) conducted experiments using four audio speeds (1.0 = normal vs. 1.5 = medium vs. 2.0 = fast vs. 2.5 = fastest rate) and auxiliary images, and the results showed that the 2.5x performance on the content recognition task was significantly lower than the other audio speeds (Ritzhaupt and Barron, 2008). Pastore (2012) processed multimedia materials by applying speed compression (0%, 25%, or 50%), and the results of the study showed that participants presented with 0% and 25% compression received similar scores and similar levels of cognitive load on both factual and problem-solving measures.

Wang (2020) found no statistically significant difference between the three video playback speeds of 1,1.1,1.2 on academic performance and no significant difference between the groups of subjects in terms of their overall satisfaction with the various video speeds, and overall positive support for the use of video acceleration in captioned video learning. Fan (2016) compared video speeds relative to three speech speeds, 140 wpm (Words Per Minute, the number of words per minute, is one of the key measures of speech rate and has been widely used in many related studies), 166–170 wpm, and 195 wpm, and found that speed changes do not significantly affect audiovisual comprehension. Qian et al. (2016) noted that in terms of delayed testing, low-experienced individuals learn better when animations are presented quickly. Ritzhaupt et al. (2015) compared subtitled and unsubtitled video groups at three levels of 1, 1.25, and 1.5, and found no significant difference in learning performance. However, satisfaction gradually decreased as the video speed increased (Ritzhaupt et al., 2015). His team further used three audio recorded at three speeds (1.0, 1.4, and 1.8) to augment a multimedia presentation as experimental material, and the results showed that there was no significant difference in the learning performance of the different speed groups (Ritzhaupt et al., 2008).

Overall, researchers hold two different views on the effect of acceleration on video learning outcomes: first, accelerated playback can promote students’ video learning, and studies by Lang et al. (2020), Feng et al. (2020), other scholars (Wang, 2020) have shown that appropriate acceleration can significantly improve students’ learning performance and satisfaction. In addition, there are also studies (Fan, 2016; Merhavy et al., 2023; Ritzhaupt et al., 2008, 2015) showing that there is no significant difference in learning effectiveness between instructional videos played at original speed and accelerated playback, but since acceleration reduces the duration of learning, it is usually considered to improve learning efficiency without inhibiting learning and thus incorporates this viewpoint as well. Secondly, accelerated playback interferes with students’ video learning. Scholars such as Duan et al. (2013), Ma et al. (2021), Sun (2023), Yang (2023), Pastore (2012), Ritzhaupt and Barron (2008) experimentally verified that accelerated playback makes learners’ cognitive load increase significantly, and the scores of maintenance test, migration test, and satisfaction with learning decrease significantly. Summarizing the above research results, it is not difficult to find that the reasons for these different research results may be manifold. Whether or not the teacher appears in the video, whether or not there are subtitles, the attributes of the country and subject matter covered in the video, the type of knowledge of the video content, and the learner’s own *a priori* level of knowledge may all have an impact on the learning effect.

### 2.3 Research hypotheses

In summary, the role of acceleration on learners’ video learning effect is not uniform, so this study adopts four indicators, namely, cognitive load, learning satisfaction, maintenance test, and migration test, to comprehensively test the effect of acceleration on video learning effect. Based on the existing literature, the research hypotheses are proposed as follows.

H1: Accelerated playback will increase learners’ cognitive load;

H2: Accelerated playback will decrease learners’ learning satisfaction;

H3: Accelerated playback decreases learners’ scores on maintenance and migration tests of video learning;

H4: The effects of video acceleration on learning outcomes differ significantly on moderating variables such as teacher appearance, subtitles, and country and subject attributes.

## 3 Methods

### 3.1 Research methods and tools

This study adopts a meta-analytic approach to systematically explore the effect of acceleration on video learning effectiveness by extracting data such as sample size, mean, and standard deviation of relevant empirical studies through literature reading, and calculating the effect size of the study, Hedges’ g. It generally involves the following steps: (1) determining the purpose of the study; (2) conducting a thorough literature search; (3) identifying an appropriate study sample; (4) defining and coding; (5) identifying statistically characterized variables; (6) entering research data; and (7) using a variety of statistical techniques to explore and present the data (Bu et al., 2021).

### 3.2 Literature search strategy

The time frame of the literature of this study is 2005.1.1–2024.11.1, and the use of adopting English and Chinese search methods, through the “multiplied speed” “video speed” “speech speed “instructional video” “video learning” “multimedia learning” and other keywords to retrieve several Chinese and English databases jointly searched, of which, the English database mainly includes SpringLink, Education Research Complete, ProQuest, ScienceDirect, Wiley Online Library and Web of Science, etc. The Chinese databases mainly include the China Knowledge Network Database and the Wanfang Database. Meanwhile, the literature backtracking method was used to further search the references, and Google Scholar and Baidu Scholar were utilized for supplementary checking.

### 3.3 Literature inclusion and exclusion

Literature selection was guided by the following seven principles: (1) only literature that used an empirical research design (e.g., randomized controlled experiment, quasi-experiment) and reported quantitative data were included, excluding reviews and theoretical discussions; (2) valid and complete data that allowed for the generation of effect sizes needed to be reported; (3) the subject of the study had to be about instructional videos, and instructional environments that did not present videos were excluded; (4) videos that encompassed all age groups of video Learners of all ages were included, but demographic characteristics of the sample had to be explicitly reported; (5) the intervention condition had to include speed adjustment of the video playback, and the acceleration method had to be explicitly stated; (6) since the purpose of the study was to explore the effect of speed on learning outcomes, at least one of the indicators of learning outcomes, such as cognitive load, satisfaction with learning, maintenance test, or migration test, was included; (7) only journal papers, conference papers, and dissertations published in Chinese and English were included, excluding unpublished gray papers, dissertations, excluding unpublished gray literature.

**FIGURE 2:**
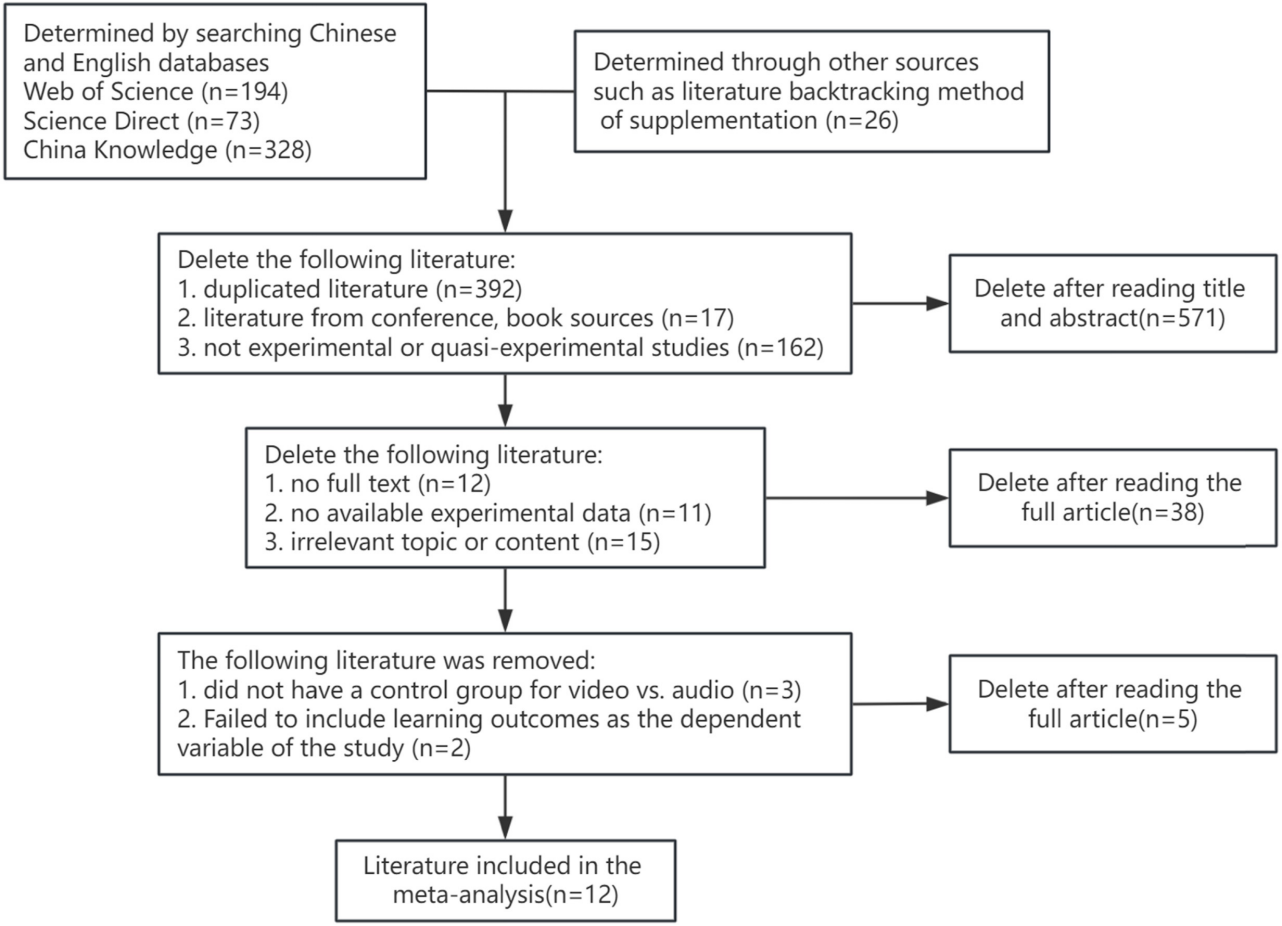
Literature screening process.

According to the literature search strategy and criteria, this study initially searched 595 journal articles, supplemented them to 621 articles through the backtracking method, removed the duplicated articles in various databases, and screened 50 articles by reading the titles and abstracts of the articles (according to the principle of literature screening); and then read the full text carefully and finally identified 12 articles that met the criteria. Among them, eight papers were in Chinese and four papers were in English. The process of literature screening is shown in Figure 2.

### 3.4 Selection of research tools, effect sizes and models

The study used Stata 17.0 software for data statistics and analysis. When dealing with continuous data with inconsistent units in different literatures, the meta-analysis generally used Cohen’s d as an indicator of effect size. However, differences in sample sizes across studies may lead to bias in estimating effect sizes by Cohen’s d, especially in potentially overestimating effect sizes in small-sample studies. Therefore, the corrected standardized mean difference Hedges’ g was chosen as a more accurate indicator of effect size in this study (Hedges, 1981). Given the variability in the selected literature in terms of participants and video materials, which may have influenced the findings, a random effects model was used in this study for the assessment of the overall effect (Yang et al., 2022a).

### 3.5 Literature coding

Literature was independently coded by two trained researchers, and for literature that met the inclusion criteria, it was coded according to the following entries (see Table 1): (1) Basic information. This included author, time, years of study, sample size, the time range (years), sample size range, participant characteristics; (2) Speed of material. Specify the speed (average speed of speech) size of the experimental materials used, and convert the speed indicators in different studies to the standard unit of WPM uniformly. Including the original speed of speech of the video (control group), adjusted speed of speech (experimental group) with the acceleration multiplier (based on the control group); (3) Outcome variables. Learning outcome indicators were coded, according to cognitive load, learning satisfaction, maintenance test (the degree of memorization and recognition of the learning material) and migration test (migration ability). In case of coding disagreements, a third senior researcher intervened to negotiate and eventually reach a consensus.

**TABLE 1:** Accelerated coding table for meta-analysis related to video learning.

References	The time range (years)	k	Sample size range	Participant characteristics	Times faster	Control group speed of speech (wpm)	Experimental group speed of speech (wpm)	Maintenance test	Migration test	Learning satisfaction	Cognitive load
Duan et al., 2013	< 1	26	Small sample	University students	Acceleration	–	–	−0.26	0.8	–	–
Duan et al., 2013	< 1	26	Small sample	University students	Acceleration	–	–	−0.31	−0.36	–	–
Fan, 2016	< 1	48	Medium sample	University students	1.16	140	166–170	−1.62	–	–	–
Fan, 2016	< 1	47	Medium sample	University students	1.2	166–170	195	−1.17	–	–	–
Wang, 2020	< 1	60	Medium sample	University students	1.1	166	183	−0.05	–	−0.02	–
Wang, 2020	< 1	60	Medium sample	University students	1.2	166	199	−0.12	–	0.01	–
Wang, 2020	< 1	60	Medium sample	University students	1.1	166	183	0.04	–	−0.02	–
Wang, 2020	< 1	60	Medium sample	University students	1.2	166	199	−0.04	–	0.04	–
Wang, 2020	< 1	60	Medium sample	University students	1.1	166	183	−0.02	–	−0.05	–
Wang, 2020	< 1	60	Medium sample	University students	1.2	166	199	−0.04	–	−0.06	–
Feng et al., 2020	< 1	20	Small sample	University students	1.25	170	212	0.51	0.32	–	–
Feng et al., 2020	< 1	20	Small sample	University students	1.5	170	255	−1.28	−1.02	–	–
Feng et al., 2020	< 1	22	Small sample	University students	1.25	170	212	−0.39	−0.67	–	–
Feng et al., 2020	< 1	21	Small sample	University students	1.5	170	255	−0.21	−0.03	–	–
Feng et al., 2020	< 1	57	Medium sample	University students	1.5	168	252	0.02	−0.34	–	−0.03
Feng et al., 2020	< 1	57	Medium sample	University students	1.5	170	255	−0.58	−0.2	–	−0.13
Ma et al., 2021	< 1	42	Medium sample	University students	1.5	–	–	−1.17	−0.39	0.41	–
Ma et al., 2021	< 1	40	medium Sample	university students	2	–	–	−1.79	−0.81	1.06	–
Yang, 2023	< 1	39	Medium sample	School children	1.5	200	300	−0.24	–	–	−0.05
Yang, 2023	< 1	38	Medium sample	School children	2	200	400	−1.03	–	–	−1.12
Sun, 2023	< 1	32	Medium sample	University students	1.5	193	289	−1.2	−1.44	–	−1.78
Ritzhaupt and Barron, 2008	< 1	75	Medium sample	University students	1.5	150	225	−0.32	–	0.79	–
Ritzhaupt and Barron, 2008	< 1	75	Medium sample	University students	2	150	300	−0.19	–	1.41	–
Ritzhaupt and Barron, 2008	< 1	76	Medium sample	University students	2.5	150	375	−0.78	–	1.84	–
Ritzhaupt and Barron, 2008	< 1	75	Medium sample	University students	1.5	150	225	−0.2	–	0.96	–
Ritzhaupt and Barron, 2008	< 1	75	medium Sample	university students	2	150	300	−0.13	–	1.3	–
Ritzhaupt and Barron, 2008	< 1	77	Medium sample	University students	2.5	150	375	−0.92	–	2.61	–
Ritzhaupt et al., 2008	< 1	123	Large sample	University students	1.4	150	210	−0.1	–	−0.18	–
Ritzhaupt et al., 2008	< 1	120	Large sample	University students	1.8	150	270	0.14	–	0.28	–
Pastore, 2012	< 1	50	Medium sample	University students	1.33	164	219	0.06	–	–	−0.01
Pastore, 2012	< 1	52	Medium sample	University students	2	164	328	−0.37	–	–	−0.54
Pastore, 2012	< 1	52	Medium sample	University students	1.33	164	219	−0.44	–	–	−0.45
Pastore, 2012	< 1	50	Medium sample	University students	2	164	328	−1.63	–	–	−1.53
Ritzhaupt et al., 2015	< 1	47	Medium sample	University students	1.25	167	209	−0.07	–	0.49	–
Ritzhaupt et al., 2015	< 1	47	Medium sample	University students	1.5	167	251	0.24	–	−3.37	–
Ritzhaupt et al., 2015	< 1	49	Medium sample	University students	1.25	167	209	−0.44	–	0.46	–
Ritzhaupt et al., 2015	< 1	47	Medium sample	University students	1.5	167	251	−0.04	–	−3.72	–

## 4 Results

### 4.1 Main effects test

In order to examine the effect of speeding up on video learning effectiveness, this study conducted a main effect test from four dimensions, namely, cognitive load, learning satisfaction, maintenance test and migration test, as shown in Table 2.

**TABLE 2:** Main effect test of the effect of speeding up on learners’ video learning effectiveness (RE model).

Outcome variables	K	*N*	g	95% CI
Cognitive load	9	427	0.59***	(0.17, 1.01)
Learning satisfaction	20	1,328	−0.24***	(−0.85, 0.37)
Maintenance test	37	1,985	−0.41*	(−0.58, −0.24)
Migration test	11	363	−0.50	(−0.75, −0.25)

*Represents different significance levels (*p < 0.05, ***p < 0.001).

In terms of cognitive load, nine studies included this variable and all of them showed that acceleration increases cognitive load. The main effects test results found a mean effect size of 0.59 (g = 0.59***), a moderate and extremely significant effect size, with both the upper and lower 95% CI limits being greater than 0, indicating that the effect was statistically significant, which is conducive to supporting the experimental hypothesis H1. In terms of learning satisfaction, there were 20 studies that included this variable, and the results of seven of them found that acceleration decreases the learner’s learning satisfaction. The main effects test found a negative mean effect size of 0.24 (g = −0.24***), a medium and extremely significant effect size, but the 95% CI interval overlaps with 0. The possibility that the effect is due to random variation cannot be ruled out, and it needs to be further determined whether the effect is real or not; for maintenance test scores, there were 37 studies that included the variable, and the results of 31 of the experiments found that acceleration would reduce maintenance test scores. The main effects test found a negative mean effect size of 0.41 (g = −0.41*), a moderate and statistically significant effect size, with upper and lower 95% CI’s less than 0; for migration test scores, there were 11 studies that included this variable, with nine of them finding that acceleration decreased migration test scores. The main effects test found a negative mean effect size of 0.50 (g = −0.50), a moderate but not significant effect size, and both the upper and lower 95% CIs were less than 0, which favored support for experimental hypothesis H3.

### 4.2 Heterogeneity test

In this study, heterogeneity tests were conducted separately for four outcome variables, including cognitive load, as shown in Table 3, and it was found that the Q-tests for the remaining three types of outcome variables, cognitive load, learning satisfaction, and test maintenance, except migration test, were all significant, which indicated that the combined effect sizes of the migration test had good homogeneity, and the other three types of combined effect sizes showed significant heterogeneity.

**TABLE 3:** Heterogeneity test for the effect of acceleration on learners’ video learning outcomes (RE model).

Outcome variables	Q	df	*P*	I2	τ2	H2
Cognitive load	32.24	8	< 0.001	77.76%	0.32	4.50
Learning satisfaction	274.33	19	< 0.001	96.36%	1.85	27.44
Maintenance test	111.78	36	< 0.001	70.48%	0.19	3.39
Migration test	14.53	10	0.15	25.72%	0.05	1.35

For cognitive load and learning satisfaction, the I2 values were 77.76% and 96.36%, respectively, indicating that the variation due to the true differences in effect sizes in the two outcome variables accounted for 77.76% and 96.36% of the total variation, respectively; and for the maintenance test and the migration test, the I2 values were 70.48% and 25.72%, respectively, indicating that the variation due to the true differences in effect sizes in the two outcome variables accounted for variation accounted for 70.48% and 25.72% of the total variation, respectively. Except for the migration test, the heterogeneity of the other three outcome variables in this study was high, so it is reasonable to use the random effects model. In addition, the heterogeneity of the effect sizes also implies that there may be potential moderating variables for the effect of video acceleration on learning outcomes, and it is necessary to determine whether there are differences in effect sizes between subgroups through moderated effects tests.

### 4.3 Publication bias test

**FIGURE 3:**
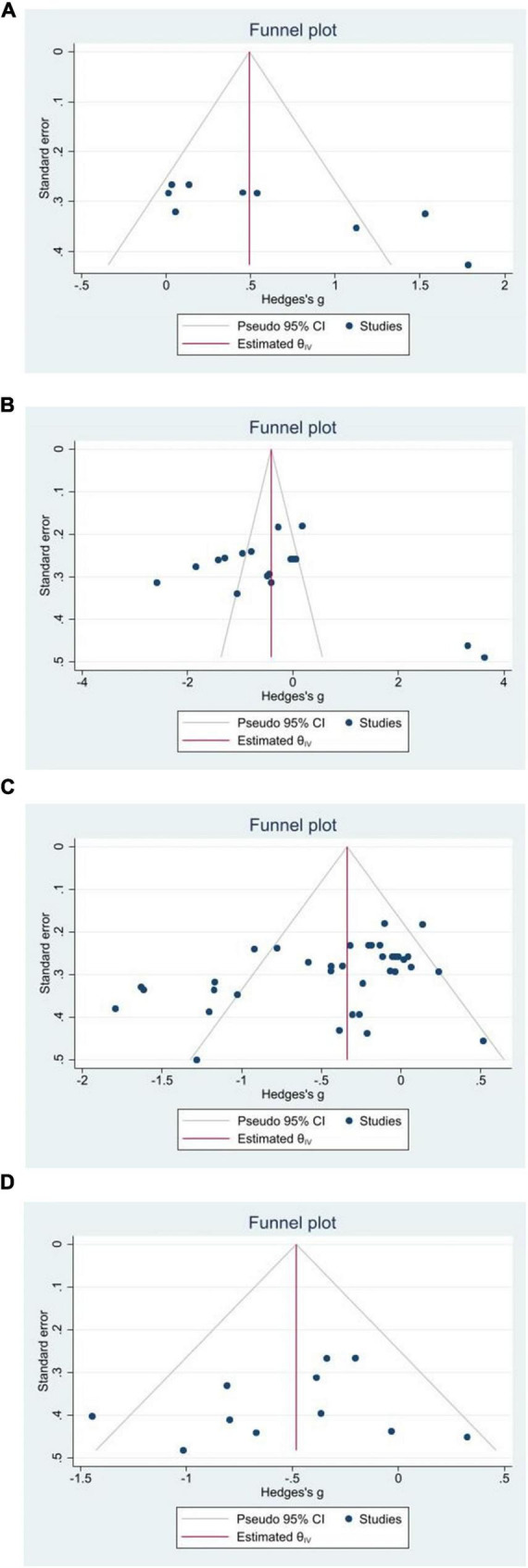
Funnel plot of publication bias evaluation. **(A)** Cognitive load. **(B)** Learning satisfaction. **(C)** Maintenance test. **(D)** Tables.

Funnel plot with cut and fill and regression based Egger’s test were used to assess publication bias in this study. As shown in the funnel plot in Figure 3, the scatters of the migration test were roughly symmetrically distributed, while the scatters of the cognitive load, learning satisfaction, and maintenance tests had obvious missing corners, which initially suggests that there may be publication bias in the cognitive load, learning satisfaction, and maintenance tests. As shown in Table 4, from the Egger linear regression, the index values of cognitive load, learning satisfaction, and maintenance test were 10.945, 11.852, and −2.911, respectively, which were not close to 0, and the 95% CI did not include 0, and the *p*-values were all less than 0.05, indicating that there might be publication bias in cognitive load, learning satisfaction, and maintenance test; however, in the migration test, the index values were −1.236, −1.236, −1.236, and −1.236, respectively. However, in the migration test, the value of this index is −1.236, 95% CI including 0, *p*-value is more than 0.05, indicating that the possibility of publication bias in the migration test is smaller. In response to the differences in the above test results, this study further used the cut-and-patch method to cut and patch the literature on the left and right sides of the effect size, and found that the effect was still significant. In summary, the cognitive load, learning satisfaction and maintenance tests may have some publication bias, and the migration test is less likely to have publication bias.

**TABLE 4:** Publication deviation test.

Outcome variables	Egger’s intercept	SE	95% CI	*P*
Cognitive load	10.945	3.014	(5.039, 16.852)	< 0.05
Learning satisfaction	11.852	3.540	(4.913, 18.791)	< 0.05
Maintenance test	−2.911	1.146	(−5.157, −0.664)	< 0.05
Migration test	−1.236	1.731	(−4.669, 2.197)	> 0.05

Specifically, in the case of publication bias in cognitive load, learning satisfaction and maintenance tests, the generalizability of the findings may be significantly affected. Publication bias may lead to an overestimation of the effect sizes, causing us to incorrectly assume that stronger effects of accelerated video learning exist on cognitive load, learning satisfaction, and knowledge maintenance. This means that conclusions about “the relationship between accelerated video learning and cognitive load, learning satisfaction, and maintenance of tests” based on the current data may not be reproducible in a broader research context. In contrast, the low likelihood of publication bias for the migration test suggests that the findings for this metric are relatively robust and more generalizable and reliable. This suggests that when assessing the effectiveness of accelerated video learning, the migration test can provide more objective evidence and can be used as one of the key indicators of learning effectiveness. However, even if the publication bias of the migration test is small, the influence of other potential biases cannot be completely excluded, and caution is still needed when interpreting the study results.

### 4.4 Examination of the effect of moderating variables

The study conducted a detailed analysis on whether four variables, namely, whether the teacher appeared in the material, whether there were subtitles, the country where the experiment was conducted and the attributes of the material discipline, played a moderating effect on video acceleration, and the results of the study are shown in Table 5.

**TABLE 5:** Moderating effect test for video acceleration.

Outcome variables	Moderator variable		k	g	95% CI	I2	z	*P*	Q_*B*_	*P*
Cognitive load	Teachers in the spotlight	Yes	2	0.538	(0.074, 1.003)	80.2%	2.270	0.023	7.11	0.029
		No	5	0.702	(0.432, 0.973)	80.0%	5.094	< 0.001		
	Country	China	5	0.421	(0.148, 0.694)	78.8%	3.026	0.002	0.59	0.441
		United States	4	0.495	(0.298, 0.692)	76.6%	3.953	< 0.001		
Learning satisfaction	Subtitles	Yes	6	0.466	(0.230, 0.703)	94.6%	3.870	< 0.001	83.63	< 0.001
		No	4	−0.199	(−0.469, 0.070)	0	−1.450	0.147		
	Country	China	8	−0.109	(−0.296, 0.079)	31.6%	−1.132	0.257	14.93	< 0.001
		United States	12	−0.401	(−0.516, −0.285)	95.6%	−7.747	< 0.001		
	Subject attributes	Natural sciences	8	−0.109	(−0.296, 0.079)	31.6%	−1.132	0.257	14.93	< 0.001
		Humanities and social sciences	12	−0.401	(−0.516, −0.285)	95.6%	−7.747	< 0.001		
Maintenance test	Teachers in the spotlight	Yes	4	−1.000	(−1.323, −0.676)	67.5%	−6.055	< 0.001	19.04	< 0.001
		No	15	−0.340	(−0.467, −0.212)	66.4%	−5.225	< 0.001		
	Subtitles	Yes	6	−0.017	(−0.219, 0.185)	0.000	−0.165	0.869	16.38	< 0.001
		No	4	−0.179	(−0.427, 0.070)	40.6%	−1.408	0.159		
	Country	China	21	−0.420	(−0.555, −0.284)	70.5%	−6.069	< 0.001	2.46	0.116
		United States	16	−0.339	(−0.429, −0.249)	63.8%	−4.456	< 0.001		
	Subject attributes	Natural sciences	21	−0.344	(−0.476, −0.212)	67.4%	−5.094	< 0.001	1.40	0.497
		Humanities and social sciences	14	−0.314	(−0.441, 0.186)	71.9%	−4.813	< 0.001		
Migration test	Subtitles	Yes	2	0.349	(−0.259, 0.958)	5.1%	1.126	0.260	0.62	0.732
		No	2	0.301	(−0.344, 0.946)	75.8%	0.915	0.360		

#### 4.4.1 Moderating effect of teacher’s appearance

This study categorized the sample into with and without teacher appearances based on the presence of teachers in the videos of the experimental materials (see Table 5 for details). In terms of cognitive load, teacher presence significantly moderated acceleration (*p* = 0.029 < 0.05), and the difference between the effect of teacher presence and no teacher presence on cognitive load was statistically significant. The effect value for no teacher screen time (g = 0.701) was greater than the effect value for screen time with a teacher (g = 0.538), indicating that acceleration was used to produce a more pronounced effect for videos without teacher screen time. The effect of acceleration on cognitive load was significant when there was teacher screen time (*p* = 0.023 < 0.05) and extremely significant when there was no teacher screen time (*p* < 0.001).

On the maintenance test, teacher screen time significantly moderated the effect of acceleration (*p* < 0.001), and the difference between the effect of teacher screen time and no screen time on the maintenance test was statistically significant. The effect value was larger for the presence of a teacher’s appearance (g = −1.000) and smaller for the absence of a teacher’s appearance (g = −0.340), suggesting that acceleration was used to produce a more pronounced effect for videos with a teacher’s appearance. The reduction effect of acceleration on the maintenance test was extremely significant both with teacher appearances (*p* < 0.001) and without teacher appearances (*p* < 0.001).

#### 4.4.2 Moderating effect of subtitles

The study categorized the sample into subtitled and non-subtitled based on the presence of video subtitles on the experimental materials (see Table 5 for details). In terms of learning satisfaction, subtitles significantly moderated the effect of acceleration (*p* < 0.001), and the difference between the presence and absence of subtitles on learning satisfaction was statistically significant. The effect value of having subtitles (g = 0.466) was greater than that of not having subtitles (g = −0.199), indicating that acceleration was used to produce a more pronounced effect for videos with subtitles. When subtitled (*p* < 0.001), the effect of acceleration on learning satisfaction was extremely significant; when not subtitled (*p* = 0.149), acceleration may reduce learning satisfaction, although the results were not significantly different.

On the maintenance test, subtitles significantly moderated the effect of acceleration (*p* < 0.001), and the difference between the effect of the presence or absence of subtitles on the maintenance test was statistically significant. The effect value for no subtitles (g = −0.170) was significantly larger than the effect value for subtitles (g = −0.017), suggesting that acceleration produced a more pronounced effect for videos without subtitles. Acceleration may reduce maintenance test scores both with (*p* = 0.869) and without subtitles (*p* = 0.159), but the results are not significantly different.

On the migration test, the between-group difference for subtitles was not statistically significant (*p* = 0.732), suggesting that acceleration produced a stable effect on the video migration test with and without subtitles. Specifically, the effect value with subtitles (g = 0.349, *p* = 0.260) is very close to the effect value without subtitles (g = 0.301, *p* = 0.360), indicating that there is no significant difference in the effect of the presence of subtitles on the migration test.

#### 4.4.3 Moderating effect of country

The study divided the sample into China and the United States based on the country where the experimental setting was located. In terms of cognitive load, the between-group difference in countries is not statistically significant (*p* = 0.441), indicating that the effect of acceleration on the migration test produced by acceleration in different country environments is stable (see Table 5 for details). Specifically, the effect value for China (g = 0.421, *p* = 0.002) was similar to that of the United States (g = 0.495, *p* < 0.001) and was at a moderate level, suggesting that acceleration had a moderate effect on cognitive load across countries and both reached a significant level.

In terms of learning satisfaction, country significantly moderated the effect of acceleration (*p* < 0.001), and the difference in the effect of different countries on learning satisfaction was statistically significant. The effect value for the United States (g = −0.401) was greater than that for China (g = −0.109), suggesting that acceleration produced a more pronounced effect in the United States setting. In the United States setting (*p* < 0.001), acceleration had an extremely significant effect on reducing learning satisfaction; in the Chinese setting (*p* = 0.257), acceleration may have weakly reduced learning satisfaction, although the results were not significant.

On the maintenance test, the between-group effect of country was not significant (*p* = 0.116), suggesting that acceleration produces a stabilizing effect on the maintenance test in different country settings. Specifically, the effect value for China (g = −0.420, *p* < 0.001) was similar to that of the United States (g = −0.339, *p* < 0.001) and was at a moderate level, suggesting that video acceleration had a moderate effect on reducing maintenance tests across countries and that both reached a significant level.

#### 4.4.4 Moderating effect of disciplinary attributes

The study categorized the sample into natural sciences and humanities and social sciences based on the disciplinary attributes of the experimental materials (see Table 5 for details). Subject attributes significantly moderated the effect of acceleration on learning satisfaction (*p* < 0.001), which was statistically significant. The effect value for humanities and social sciences (g = −0.401) was greater than that for natural sciences (g = −0.109), suggesting that acceleration used for videos in humanities and social sciences produced a more significant effect. For humanities and social sciences (*p* < 0.001), the effect of acceleration on the reduction of learning satisfaction was extremely significant; for natural sciences (*p* = 0.257), acceleration may slightly reduce learning satisfaction, although the results do not differ significantly.

On the maintenance test, the between-group effect of subject attributes was not significant (*p* = 0.497), suggesting that acceleration produces a stable effect on the maintenance test across subjects. Specifically, the effect values for natural sciences (g = −0.344, *p* < 0.001) and humanities and social sciences (g = −0.314, *p* < 0.001) were similar and at a moderate level, suggesting that video acceleration across disciplines had a moderate effect on reducing maintenance tests and both reached a significant level.

## 5 Discussion

### 5.1 The effect of acceleration on cognitive load

From the results of the main effects test, the increase in cognitive load by accelerated playback of instructional videos reached a highly significant level (g = 0.59, *p* < 0.001), and the hypothesis H1 was valid, i.e., accelerated playback significantly increased the cognitive load of learners compared to original speed playback. This is in full agreement with the results of previous studies.

Cognitive Load Theory states that an individual’s cognitive load refers to the workload of the cognitive system when performing a specific task, which can be distinguished as intrinsic cognitive load, extrinsic cognitive load, and related cognitive load. While learning, intrinsic cognitive load is fixed for tasks at a specific level of knowledge, external cognitive load is an additional load beyond the internal cognitive load, which is mainly caused by poorly designed instruction, and relevant cognitive load refers to the load associated with facilitating the process of schema construction and schema automation (Paas et al., 2004). Accelerated playback of instructional videos does not change the interaction between the nature of the learning material and the expertise of the learner, nor does it affect the generation of schema construction, but it excessively increases the amount of information per unit of time, which exceeds the limited capacity of the human working memory, resulting in a cognitive overload in the visual and auditory channels, i.e., it leads to an increase in the extrinsic cognitive load (Wang et al., 2013). Thus video acceleration, an inappropriate presentation, has a significant impact on cognitive load.

### 5.2 Effect of acceleration on learning satisfaction

From the results of the main effects test, the reduction of learning satisfaction by instructional video acceleration reached a significant level (g = −0.24, *p* < 0.001), and hypothesis H2 was established. This is consistent with the results of seven previous studies. The probable reason for this is that the video speech rate of 183–251 WPM selected for the sample of these seven experiments is on the slow side overall, which may lead to learners’ impatience with the overly slow learning process and instructor’s teaching, and therefore a significant decrease in learning satisfaction. The inconsistent results of the other 13 studies may be due to some differences in satisfaction with video learning due to individual learner differences and material factors such as instructor’s rate of speech and difficulty of video content (Wang, 2020). Taken together, it seems that the present study is consistent with the findings of a few studies, but the moderate and significant effect size also reflects the significance of learners’ self-regulation of video playback speed in the aspect of satisfaction with video learning.

### 5.3 Effects of acceleration on maintenance and migration tests

Hypothesis H3 is partially true as the effect of speeding up on the reduction of the maintenance test (g = −0.41, *p* < 0.05) is significant and the effect on the migration test is not significant. That is, compared with the original speed of playback, accelerated playback of instructional videos will hinder the learners’ memorization and recognition effects, but will not hinder the learners’ migration ability.

Combined with the analysis of the theoretical model constructed (as shown in Figure 1), the possible reasons are as follows: first, in the cognitive process of video learning, information is selected through receptors such as the eyes and ears to form sensory memory and organized and processed into working memory (Chen, 2007). Excessively fast video speed means that the density of information coming into the visual and auditory channels is too high for the receptors to receive all the information and exceeds the limited capacity of working memory, which makes the brain unable to store information in time, leading to low memory and recognition effects of learners during maintenance tests. Secondly, Cognitive Capacity states that attention is the ability to focus attention and resources on stimuli of interest, the ability to filter irrelevant or intrusive stimuli, and is important for learners in selecting, processing, and attending to information (Sternberg and Mio, 2008). Learners have limited attention, and video acceleration conditions place excessive demands on perceiving and capturing information beyond an individual’s attentional capacity, resulting in inadequate perception and processing of video information, which affects literacy.

A moderate but non-significant effect size was found for acceleration on migration test scores (g = −0.50, *p* > 0.05), suggesting that video speed has a greater impact on shallow literacy and recall of learning content relative to migration ability. The possible reason for this is that changes in video speed are more likely to be reflected in affecting learners’ perceptual processing of relevant learning information, whereas migration tests often require learners to reason about the interactions between different elements of the learning material and construct more complex mental models (Yang et al., 2022b), and video speed has a limited effect in this regard. In addition, the literature adopted for this meta-analysis has a small number of independent effect sizes (K = 11) for migration tests, which may lead to systematic biases between the calculated composite effect sizes and the actual effect sizes.

### 5.4 Effects of moderating variables on learning outcomes

#### 5.4.1 Teacher appearances

In terms of teacher appearances, acceleration had an increase in cognitive load for both videos with and without teacher appearances. Among them, the enhancement effect is stronger for no teacher appearances. This may be because when the teacher’s image is presented in the teaching video, it will lead to information redundancy (Sweller, 1988), and the original cognitive load is higher, and acceleration will reduce the information processing time and thus increase the overall cognitive load; whereas in the case of no-teacher-appearance, the learner does not have the cognitive load due to the teacher’s image, and the original cognitive load is even lower, so the increase in the overall cognitive load after acceleration is even more pronounced.

For the maintenance test, acceleration had an impeding effect on the maintenance test for both videos with and without the teacher’s appearance. The hindering effect was stronger for videos with teacher appearances. Attention runs through the entire learning process, in which the learner, under the action of external stimuli, first generates attention, selects information related to the current learning task through the directionality of attention, and at the same time utilizes the focus of attention to ignore other irrelevant stimuli, activates the relevant original knowledge in the long-term memory, and ultimately completes the fusion and construction of the old and new knowledge (Yuan et al., 2020). When there is no teacher in the scene, learners’ attention is focused on the learning material, and the frequency of learning material change becomes faster after acceleration, even if the full attention will have an impact on the acceptance and processing of information, while when there is a teacher in the scene, with the acceleration of the speed of the teacher’s image changes more rapidly, and its expression, gaze, gestures, etc. can continuously attract the visual attention of the learners (Kleinke, 1986), compared to the learning content of the attention is greatly reduced, seriously affecting the memory and recognition of knowledge, so the acceleration of the maintenance test with teacher appearances has a stronger hindering effect. At the same time, however, it is important to note that teacher presence may have relevant signaling effects, as well as benefits in terms of social perceptions, engagement, or satisfaction (Mayer and Fiorella, 2022).

#### 5.4.2 Subtitling

In terms of subtitles, acceleration can effectively increase learning satisfaction in the subtitled condition, and acceleration may reduce maintenance test scores in the unsubtitled condition. CTML emphasizes the integration of audio-visual coded information in the brain and suggests that the simultaneous fusion of the use of these two mental channels is more efficient in information processing than a single channel (Wang et al., 2013). That is to say, the presentation form combining spoken narration and subtitles can attract learners’ attention, stimulate their interest, and be more conducive to their cognitive processing than monolingual or monotextual forms. Whereas in the accelerated condition, the requirement for learners’ information processing ability becomes higher, subtitles, as a supplement to the visual channel, greatly help learners’ overall learning (Yu et al., 2021), and therefore effectively improve learning satisfaction. It should be noted, however, that presenting both text (subtitles) and spoken language at the same time may have a redundancy effect and therefore needs to be managed (Kalyuga and Sweller, 2022). When there are no subtitles, the lack of visual representation supplements, leading to learners’ difficulties in information processing, and therefore acceleration in the no-subtitle condition may reduce maintenance test scores.

#### 5.4.3 Country

Country-wise, acceleration significantly reduced learning satisfaction in the American setting, probably because the American sample selected for the study had a greater degree of acceleration in the experimental design, with a minimum of 1.25x speed and a maximum of 2.5x speed, while the Chinese sample had a minimum of 1.1x speed and a maximum of only 2x speed. The high video playback speed on the one hand gives learners more information processing pressure, causing students to miss important details or concepts in depth, and on the other hand, its rapid video pace makes students feel rushed and anxious rather than enjoying the learning process, thus significantly reducing learning satisfaction.

#### 5.4.4 Subject attributes

In terms of subject attributes, there are two possible reasons for the significant effect of accelerating the reduction of learning satisfaction in humanities and social sciences. One is that humanities and social sciences usually involve the understanding and appreciation of language, literature, history, and other aspects (Li, 2012), accelerated video causes learners to have difficulty in appreciating the beauty of language and content, thus reducing their learning satisfaction, and the second is that in the learning of humanities and social sciences, it is valued that the in-depth experience of the content and in-depth thinking (Zhang, 2008), accelerated so that the learners’ thinking becomes superficial, and they are unable to think deeply about the connotations of humanities and social sciences. Therefore acceleration reduces the satisfaction of learning humanities and social sciences significantly. In summary, hypothesis H4 is valid.

## 6 Conclusion

The following conclusions were drawn from this study: (1) Accelerated playback of instructional videos increases learners’ cognitive load, which is not conducive to the effects of memorization and recognition of learning materials, as evidenced by lower maintenance test scores, as well as a decrease in learning satisfaction. (2) The effect of acceleration on learners’ migration test scores was not significant. (3) Teacher appearances play a moderating role in the process of video acceleration affecting cognitive load and maintenance tests, and subtitling, country, and subject attributes play a moderating role in the process of video acceleration affecting learning satisfaction.

This study mainly has the following shortcomings: first, the meta-analysis puts strict requirements on the quality of the empirical research literature, and there is a scarcity of empirical data for the research on instructional video speed, which undoubtedly increases the difficulty of the study. Some of the empirical studies chose silent animation as the material in the experimental process, which was categorized in the category of multimedia rather than video, resulting in some of the literature not being included in the sample set, which cut down the number of literature included in the meta-analysis and the number of effect values, which in turn affected the statistical validity of the research results. In particular, the migration test included only 11 studies, which may affect the reliability of the results. A small sample reduces the power of the statistical test while amplifying the effect size estimation error, leading to a lack of stability of the conclusions. A limited sample still constrains its external validity and affects inferences to broader educational scenarios. It is recommended that subsequent studies enhance the statistical efficacy by expanding the sample size and using multicenter data to enhance the generalizability of the findings. Second, video playback speed adjustment was viewed as a complex and difficult to clearly define research dimension. Because the concept of accelerated playback is inherently relative, there is variability in the speed of speech across videos and a lack of a uniform frame of reference. Playback speed should be regarded as a continuous variable. For the sake of meta-analysis, this paper simplifies the speed of playback into two categories: “accelerated” and “original,” which undoubtedly brings methodological limitations and may potentially bias the results of the study. Future research needs to consider more refined categorization methods to improve the accuracy and generality of the analysis.

With the widespread popularity of online education and video teaching, the practice and research of instructional videos have become increasingly rich and in-depth. Researchers still need to work on determining the appropriate video speed or speech rate to facilitate learners’ cognitive processing and ultimately improve learning. Given the publication bias in indicators such as cognitive load and learning satisfaction, it is recommended to adopt a step-by-step acceleration strategy in video design. First, explain the core concepts at the original speed, and then moderately accelerate the review content. At the same time, technologies such as eye movement tracking and real-time feedback can be used to dynamically monitor the cognitive load of learners and adjust the video rhythm in a timely manner. In response to the problem of a small sample size in the migration test, when designing the video evaluation part, educators should add diverse migration tasks, such as case analysis and scenario simulation, to more comprehensively evaluate the learning effect. Future research on instructional video can consider expanding from the following aspects: First, using advanced technologies such as eye tracking, electroencephalography, and emotion recognition to comprehensively assess the effect of speed on learners’ cognitive neural activities and thus affect their learning. Secondly, since there are more studies on learners’ knowledge learning and less on motor skills and attitudes, with the gradual advancement of the concept and practice of human-centered and holistic development education, we can pay attention to the research on video design at the level of motor and attitudes in the future. Third, with the promotion of new forms of educational resources, research on panoramic video is still in its infancy, and in the future, it is necessary to expand the research boundaries to further analyze the impact of the speed of panoramic video on learning and compare it with flat video.

## Data Availability

The datasets presented in this study can be found in online repositories. The names of the repository/repositories and accession number(s) can be found in the article/supplementary material.
